# Progressing implementation of behavior change frameworks for digital health interventions: challenges and ways forward

**DOI:** 10.1093/tbm/ibaf069

**Published:** 2025-12-12

**Authors:** Ananya Ananthakrishnan, Cen Cong, Victoria Riccalton, Edward Meinert

**Affiliations:** Translational and Clinical Research Institute, Newcastle University, Newcastle upon Tyne, United Kingdom; Translational and Clinical Research Institute, Newcastle University, Newcastle upon Tyne, United Kingdom; Translational and Clinical Research Institute, Newcastle University, Newcastle upon Tyne, United Kingdom; Translational and Clinical Research Institute, Newcastle University, Newcastle upon Tyne, United Kingdom; Department of Primary Care and Public Health, School of Public Health, Imperial College London, London, United Kingdom; Gnosis Health Limited, The Catalyst, 3 Science Square, Newcastle Helix, Newcastle Upon Tyne, United Kingdom

**Keywords:** health behavior, behavior change, digital health, digital behavior change interventionsGraphical abstractGraphical abstract

## Abstract

**Background:**

New behavior change theories, models, and frameworks (TMFs) to support digital behavior change interventions have been called for and are emerging, but low reported usage impedes progress in the field.

**Aims and objectives:**

This study aims to highlight key barriers to the implementation of digital-focused TMFs and provide recommendations to mitigate these barriers.

**Methods:**

The adoption of seven novel TMFs designed for digital behavior change interventions was analyzed based on the number of citations and the number of interventions developed using the TMFs, in comparison with the longer-established behavior change wheel (BCW) framework.

**Results:**

The uptake of the novel TMFs appears slow compared to the well-established and commonly used BCW framework, even when accounting for the recentness of the novel TMFs. Potential barriers to adoption include a lack of awareness, insufficient empirical evidence, and challenges in operationalizing these models. Researchers creating these TMFs could mitigate these barriers by improving their design (e.g. by making them more adaptable) and reporting and developing, and testing interventions based on their TMFs to start building an evidence base.

**Conclusions and future research:**

New TMFs with a focus on digital technologies have the potential to improve the efficacy and outcomes of digital behavior-change interventions, but their accessibility, evidence base, and reporting must improve to facilitate their adoption in intervention design. Further review work comparing the novel TMFs with traditional frameworks and a repository to guide researchers in framework selection could also help with faster uptake.

## Teaser

Existing behavior change theories are not being used widely enough in developing digital interventions. We analyses why this might be and provide recommendations to improve uptake.

Implications **Practice:** There is a substantial gap between the development of theories tailored for digital behavior change interventions, so it is important for practitioners to consider behavior change theory while developing interventions.

**Policy:** Guidelines for the use of theories, models, and frameworks in the development of behavior change interventions would be helpful, and government investments should focus on the development of interventions grounded in theory.

**Research:** Researchers developing behavior change theories tailored for digital interventions should make these theories accessible by providing detailed strategies for their use in designing interventions, ensuring adaptability of their theories to the different applications in technology, and generating preliminary evidence to support their utility.

## Introduction

Interest in digital behavior change interventions (DBCIs), in particular mobile health (mHealth) apps, continues to grow [1]. This growth is driven by their potential to reach larger at-risk populations less resource-intensively than traditional offline methods. However, evidence for the impact of DBCIs and mHealth apps is mixed, indicating considerable room to improve their design and ultimately deliver greater patient benefit [2]. Technological advancement offers increasing interactivity and adaptive approaches, driving the development of new behavior change theories, models and frameworks (TMFs) specific to digital health interventions [3, 4]. While the three terms (theories, models, and frameworks) tend to be used together, they have distinct roles in intervention development. Theories provide high-level explanations of the mechanisms behind behavior change. Models simplify these explanations into more manageable components, and frameworks provide structures or plans as a foundation for building interventions [5]. While developing interventions, theories can define behaviors to target, models indicate how these can be applied to behavior change interventions, and frameworks help structure the creation of interventions [5, 6]. The uptake of digital health-specific behavior change TMFs by intervention designers appears slow, which may impede the advancement of the field. A time lag between TMF development and implementation is inevitable, but within the fast-moving field of digital health, quickly identifying and addressing barriers to the use of new TMFs is critical to ensure that their evolution keeps pace with technological advancement to optimize patient benefit.

While there is a strong case for continued investment in the development of DBCIs, evidence supporting their effectiveness remains limited, and efforts to improve impact on health outcomes are ongoing [7, 8]. DBCIs can have a greater impact on behavior change when aligned with behavioral science theory, but digital health designers express mixed feelings about the value of TMFs. While developers use them as a starting point and to add credibility to their work, the number of TMFs available in behavior science, their inability to go beyond conceptual explanations, and their lack of consideration of the iterative nature of digital health have been cited as reasons for not using them as comprehensive development guides [9]. This ambivalence among designers suggests that the design and accessibility of TMFs need to be improved to ensure they are fit for purpose [9].

The development of DBCIs requires multidisciplinary expertise. Digital development is typically fast-paced, iterative, and continuous, while evidence-based medical advancement takes longer and occurs in incremental steps. The need for digital health evaluation to evolve to reconcile these differences has been recognized for some time, but the acknowledgement of the same issue in DBCI development is more recent [3, 9, 10]. Well-established TMFs such as the social cognitive theory [11], the transtheoretical model [12], and the BCW framework [13] have been widely cited in both digital and nondigital contexts, but were not developed with digital interventions in mind [4, 9]. Building on these traditional TMFs, digital health-specific TMFs that could address the multidisciplinary needs of DBCI developers are emerging, but their implementation appears low; few development papers explicitly state TMFs as the bases of the approaches adopted and decisions made.

A recent scoping review by Pelly *et al.* [3] identified seven novel TMFs developed specifically to inform DBCI development and examined their theoretical foundations, stated purpose, and application to interventions. The review highlighted the growing interest in tailored TMFs for digital health contexts but did not explore their adoption in real-world practice. This commentary aims to address this gap, building on the review by outlining key barriers to the adoption of the TMFs identified in the review and suggesting critical next steps to advance the field, drawing from the consolidated framework for implementation research (CFIR) [14] to guide the perspective.

## Methods

This commentary summarized the adoption of seven digital health-specific TMFs first identified in a scoping review by Pelly *et al.* [3], and analyzed potential barriers to uptake using an implementation science approach guided by the CFIR.

To examine the adoption or uptake of the TMFs, we summarized the number of citations of the models from their publication until 16 October 2024 and compared the citation trend over the years with that of the BCW framework [13]. The BCW framework was chosen as an example as it is a longer-established behavior-change framework that has been commonly applied in both traditional behavior-change interventions and DBCIs. Citation metrics were obtained from PubMed for the first five models and the BCW, IEEE Xplore for the sixth, and SpringerLink for the last. Different databases were used to obtain citation counts for the last two models as the papers were not indexed in PubMed. Three authors with expertise in psychology and digital health then reviewed the individual papers citing the TMFs, examining the type of publication (review or original research) and the context under which the TMFs were mentioned. Due to time and resource constraints, the work of reviewing the papers was divided among the three authors, with consensus reached through discussions.

Following this, we analyzed potential barriers to implementation using the CFIR, which defines five domains—innovation characteristics, outer setting, inner setting, individuals, and implementation process—that influence the implementation of any intervention. These domains provided a structured method to group and interpret challenges observed across the literature and to explore implications that may not be obvious while reading the literature in isolation. Key themes were mapped to three CFIR domains (innovation, individual, and inner setting) through iterative reading, cross-comparison of studies, and interpretive synthesis. Initial reading and theme identification were done individually, followed by consensus meetings to align on themes and domain allocation. We examined limitations of the TMFs mentioned in the papers describing them, those citing the TMFs, and the Pelly *et al.* review [3]. For example, barriers related to the adaptability and operational clarity of the TMFs were mapped to the “innovation” domain, while the lack of awareness and mixed perceptions of usefulness related to the “individuals” and “inner setting” domains. We focused on these three domains as the data available from published literature on the reported use of the TMFs did not provide sufficient insight into the implementation processes or external policy influence.

Where relevant, we also included potential facilitators or strategies to mitigate the identified barriers, drawing on available literature as well as insights from the implementation of the BCW framework.

## Behavior Change Frameworks for DBCIs

While some DBCI developers recognize the value of applying behavior change TMFs to design their interventions, only about a third tend to use any behavior change frameworks to guide development [9], often with mixed efficacy [15]. This mixed evidence of efficacy can be attributed to the unique challenges that DBCIs face compared to nondigital interventions, such as low engagement and adherence [16], relatively low trust in technology [17], and the rapidly evolving nature of digital technologies resulting in a greater need for adaptability [18].

Among the well-established TMFs, the BCW framework has been used extensively in the DBCIs (cite). Centered around the Capability, Opportunity, Motivation, and Behaviour (COM-B) model, which explains behavior as influenced by capability, opportunity, and motivation, it guides the selection of intervention components or policy categories to bring about the change. Since its inception, numerous interventions (both traditional and digital) have used the framework to influence health behaviors in the domains of weight management, mental health, and addiction management [19–25]. The framework is often cited as having helped identify how to change the behaviors or to select intervention components [26, 27]. However, the BCW and other established behavior change frameworks were not developed for the digital context and often need to be adapted, informing DBCI development in combination with other frameworks such as User-Centered Design or Persuasive Systems Design for a better fit [3, 28, 29].

Recognizing these challenges, several novel frameworks have recently been developed to integrate digital technology with existing behavior change frameworks [3]. Unfortunately, the uptake of these novel interventions appears slow compared to the rapid advancement of technology. We demonstrate this gap using seven novel frameworks recently developed, as reviewed by Pelly *et al.* [3]. To date, this is the most recent review that summarizes the novel models and frameworks, so we draw insights from this valuable review in this study.

Out of the seven models demonstrated in Table 1, only two have 20 or more citations; and most studies citing the models use them as supporting points in their introduction or discussion sections or review the models themselves. While the studies are recent (most published less than 5 years ago) and might see an increased trend of use in later years, the high speed of technological advancements necessitates faster adoption of the digital health-specific TMFs. Two of the frameworks [33, 35] have been used to develop one intervention each, of which one intervention [36] was not directly related to the framework but retrospectively “mapped” its intervention development steps to the Iterative Framework for DBCIs [35]. The other intervention [34] *was* developed using the TUDER framework [33], but it was a protocol for an evaluation study, with no evidence yet of the intervention being effective. We reflect on the potential reasons for this distinct theory-intervention gap.

**Table 1:** Novel behavior change models and studies that cite them

ID	Name of model	Year of publication	Brief description of model	No. of citations	No. of interventions using the model	How the intervention(s) used the model
1	Adaptive decision-making framework [30]	2021	The framework maps behavior change techniques (BCTs) to action-level learning and decision-making, and reflection-level decision-making and adaptation, and provides suggestions on how to create digital health interventions to influence each of the categories.	15	0	No interventions were designed, except by the original research team where they were implicitly informed.
2	Adaptive behavioral components model [31]	2020	The model outlines five areas for intervention developers to consider—including basic behavior-change components (i.e. traditional behavior-change models), intervention, population, and individual-level characteristics, and the technology being used—but no guidance for decision-making. The model encourages developers to plan for potential changes, taking into account the dynamic nature of digital technology.	7	0	No description of use in intervention design but the original research team mentions the model when defending the limitations of a later intervention study.
3	Effort-optimized intervention model [32]	2021	The model focuses on optimizing the effort required for behavior change using a three-step approach: (i) nurturing the degree to which the behavior is considered important (salience); (ii) making the behavior as effortless as possible; and (iii) translating the activities into a sustainable commitment or habit.	12	0	No evidence of usage found.
4	Targeting, understanding, designing, evaluating, and refining (TUDER) framework [33]	2019	The model proposes four steps and a workflow to create digital health behavior-change interventions—(i) Learning about the target population and behavior-change needs; (ii) Understanding the strategies required by referring to existing strategies; (iii) Designing the intervention; and (iv) Evaluating the designed intervention and refining it based on the results and feedback gathered.	31	1	The intervention was modelled using the framework. No evidence about the intervention, as it was a protocol [34]
5	Iterative framework for digital behavior change interventions [35]	2020	The framework proposes an iterative approach to intervention development, built on the human-centred design approach. It includes five phases—predefine (understanding context and target behaviors), define (decide the intervention strategy), design (create wireframes and prototypes), develop (develop a low-fidelity intervention for testing), and deploy (first in a test environment, then in real-world settings), to be employed cyclically (i.e. results from deployment should be used to optimize the intervention).	32	1	The framework was retrospectively mapped to the intervention [36]
6	User-centric adaptive intervention model [37]	2020	The model emphasizes using technology to instigate behavior change using four stages: (i) Behavior Quantification (prioritizing target behaviors); (ii) Behavior-context Mapping (mapping healthy behaviors to different contexts, using the Transtheoretical Model); (iii) Intervention Selection (selection of types of features); and (iv) Feedback evaluation (evaluating the developed intervention).	3	0	No evidence of usage found.
7	Persuasive mobile health application model [38]	2020	The model proposes a combination of the COM-B model, the BCW framework, and the Persuasive Systems Design (PSD) model. It proposes steps to map behaviors found using the COM-B and BCW models to PSD features.	5	0	No evidence of usage found.

**Table 2:** Citation trends of models (2011–24)

Model name	2018	2019	2020	2021	2022	2023	2024
Adaptive decision-making framework (2021)	0	0	0	4	0	1	0
Adaptive behavioral components model (2020)	0	0	2	1	2	2	0
Effort-optimized intervention model (2020)	0	0	0	2	3	5	4
Targeting, understanding, designing, evaluating and refining (TUDER) framework (2019)	0	3	2	9	3	5	0
Iterative framework for digital behavior change interventions (2020)	0	1	2	5	6	2	4
User-centric adaptive intervention model (2020)	0	0	0	0	2	1	1
Persuasive mobile health application model (2020)	0	0	0	4	0	1	0

## Narrowing the Theory-Intervention Gap

We identified multiple potential barriers that might lead to the theory-intervention gap highlighted in Table 1 by mapping limitations of the digital-specific TMFs to the CFIR domains. These barriers include a lack of awareness of the DBCI-specific TMFs, a lack of evidence of their utility, and difficulties in operationalizing them in the design of DBCIs. In the following section, we suggest how future work can address them to facilitate faster uptake of the TMFs and ultimately develop better and more effective DBCIs. Using the CFIR framework allowed for a better exploration of the barriers and enabled us to offer structured, actionable pathways to improve the adoption and impact of TMFs in DBCI development.

### Lack of awareness of digital health-specific TMFs

**Figure 1:**
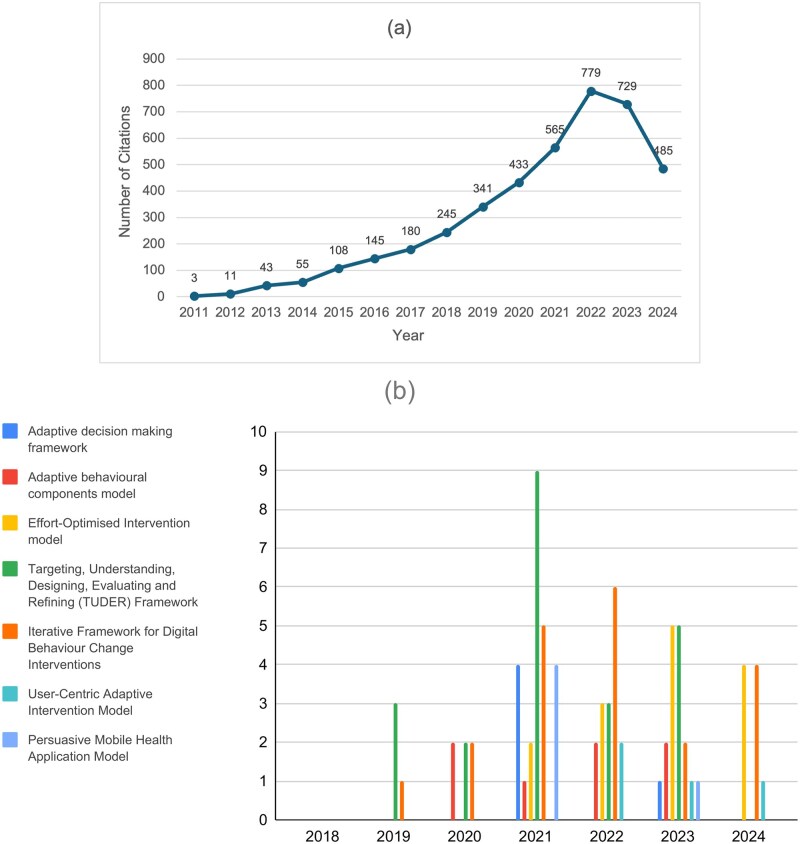
Trend comparison of citations of models (2011–24). (a) Behavior change wheel; (b) the seven digital-specific models.

The trends in citations over the years indicate a lack of awareness of novel behavior models and frameworks among researchers in the field of DBCIs. The number of citations was summarised in Table 2 and visualised in Fig. 1. The trends align with the “Knowledge and Beliefs” construct within the Individuals domain in the CFIR [14].

The BCW does not appear to have gained much attention in the first four years since its publication and gradually gained popularity in research after 2014 (see Fig. 1a). In contrast to its steady increase in popularity, the seven novel models have received both lower levels of attention and more fluctuating changes in interest (see Fig. 1b). While this might be due to the relative newness of the models, their uptake still appears slower than with the BCW, particularly considering that most papers describing the newer TMFs were only cited in passing, with only two being used to develop DBCIs. Given the rapid technological advances and high speed of digital intervention development, this might lead to interventions developed with novel digital health-specific models, which may no longer be suitable for the new technical context. To mitigate this issue and facilitate faster adoption of the novel frameworks, we provide two recommendations.

First, there is a need for more comprehensive reviews comparing the traditional behavior-change models with existing digital health-specific models. While reviews of DBCI frameworks exist [3, 39], their focus is on the digital frameworks, with a lack of comparison with traditional models. Future reviews should make it easier to understand the differences between traditional frameworks, such as the BCW and the Transtheoretical Model [12, 13], and these newer frameworks, and the contributions of the digitally focused frameworks by providing direct comparisons. Such comprehensive coverage would help researchers and DHI developers clearly visualize how the novel models and frameworks build on the traditional ones, making it easier to decide which one is a better fit for their application.

Second, a database listing all existing models might help researchers and DHI developers choose which framework would best meet their needs. The database should include potential use cases of each TMF and list their respective strengths and limitations to facilitate decision-making. Alternatively, a decision-making toolkit that provides recommendations of frameworks that could be used based on criteria such as the target population, target behavior(s), and digital technology could also save time and reduce the guesswork that might be involved in choosing one among many new models. Creating and maintaining such a database, particularly given that new TMFs might be developed or existing TMFs may be updated, could be a challenging and resource-intensive task. However, similar databases and toolkits do exist in other scientific areas, such as the dissemination and implementation models in health toolkit [40] and the Implementation Outcome repository [41]. These appear to have been funded through institutional support or project grants, although publicly available information about how they are maintained is limited. Some of the potential challenges include keeping the database and toolkit up-to-date, ensuring a balance between providing a detailed resource without making it overwhelming, and making sure the assessment of strengths and limitations is objective and unbiased. Establishing a regular review schedule of the resource, as well as allowing researchers to suggest the addition of new models, could help keep the resource updated; although the additions would need to be reviewed by experts to ensure validity. Involving potential users of the resource in the development process and testing it for usability could help ensure that it is user-friendly. To keep the assessment of the TMFs unbiased, transparent evaluation criteria should be decided, and the assessment should be undertaken by at least two independent reviewers.

### Lack of evidence from interventions

By exploring studies that cited the above novel models, we have found that researchers often chose to use the more established and influential behavior models instead (such as the COM-B model [13]), even when they were aware of novel behavior models and frameworks for DBCIs. This is reflected in studies that cite the novel TMFs in their introduction or discussion sections, yet rely on established models when developing interventions, and in the discrepancy between papers that reference TMFs and those that apply them in intervention design (Table 1). One possible challenge for the adoption could be a lack of robust empirical evidence demonstrating the effectiveness and real-world applicability of these newer models. In most cases, the original researchers who proposed the models have not provided case studies on how these models can be used in the design of digital health interventions. This lack of supporting evidence may limit the subsequent researchers’ understanding of the models and how they could conduct additional studies to replicate the process and make improvements. Such a cycle of exploration would generate evidence on the validity and reliability of the models, driving their evolution. In contrast, more established TMFs such as the BCW have been applied to a wide range of interventional studies, making them more credible and potentially contributing to continued adoption [42–44].

To address this issue, we recommend that the authors developing the digital health-specific TMFs (who best understand them) design interventions based on these TMFs and objectively demonstrate their utility, efficacy, and impact. Ideally, such examples should include clear methodological detail, links to the desired behavior change outcomes, and discussion of practical implementation challenges. Such demonstrations could generate trust in the frameworks among other researchers and contribute to their understanding of how the model or framework can be implemented in the real world, allowing other DHI developers to replicate the process. They also open up opportunities for other researchers to adapt the TMFs based on the generated evidence, thus enabling researchers to further build on their work.

## Difficulties in Operationalization

The novel TMFs described in this study have either combined elements from multiple well-established frameworks (including behavior-change and digital TMFs) or built on a single TMF, or both. One major challenge, as mentioned in one of the studies that considered the novel models, is that they are often difficult to operationalize in the design of digital health interventions [45]. The models may have integrated multiple concepts or elements based on several theories, but there is a lack of detailed guidance on how to implement these concepts or elements in practice. With concepts alone, it would be challenging to apply them to future studies. Additionally, the context where the models could be applied may not be clear enough for researchers to apply them across diverse intervention settings. The effort-optimized Intervention model, for example, highlights user engagement and competing demands in digital interventions [32]. Another example is the adaptive behavioral components model, which accounts for changing contexts but lacks the specificity required to make it easily implementable [3, 31]. It has received some attention from scholars when they analyzed the recruitment effort in randomized controlled trials, but it was often not considered while designing the trials nor in the design of other digital interventions. The types of digital interventions (e.g. education and training techniques) and the platforms for their delivery (e.g. mobile applications and websites) may affect how the novel models can be applied, as the elements included in the original studies may not apply to a new intervention.

Therefore, it is important to be more specific about context and develop more flexible, generalizable frameworks so that researchers can confidently adopt these newer approaches in their design of interventions. Models integrating behavior-change theory with DBCIs must reach the right balance between adaptability (i.e. easily applicable to different contexts and technologies) and specificity (i.e. specific to health behavior-change contexts). Future frameworks could reach this balance by combining adaptable and scalable components with clear guidance for specific applications. For example, including guidelines for adapting a model to different digital technologies could improve adaptability without compromising its relevance.

A better balance between adaptability and specificity is achieved by traditional frameworks such as the BCW and the Transtheoretical Model. The BCW, for example, is broadly applicable across health domains but offers clear pathways for operationalization through actionable guides, an online toolkit, and a comprehensive taxonomy and ontology [46–49]. Similarly, the transtheoretical model provides a general, stage-based model that is applicable to a range of behaviors (e.g. exercise, diet, medication adherence, etc.) across contexts and settings, while also providing tailored intervention strategies for each stage [12]. This successful balance is a potential reason for their widespread use in intervention design, although they were not designed with digital contexts in mind and do not account for the specific challenges of rapid iteration, real-time data use, and interface variability. While the novel TMFs attempt to account for the broad range of digital contexts, they have not yet reached similar levels of operational clarity, which is a crucial area of future development.

## Conclusion

With reference to seven recent TMFs created specifically for DBCI design and development, we have highlighted a substantial gap between theory development and their practical application in intervention development. Our study indicates that the uptake of the novel TMFs is slow, given the fast-evolving digital landscape, as well as when compared to established frameworks such as the BCW. Using the CFIR as a guide, we analyzed barriers to uptake, highlighting potential reasons for this challenge mapping to CFIR constructs within the innovation, inner setting, and individuals domains. From an implementation science perspective, it is important to understand and address this gap and, to this end, this study recommends empirical evidence to demonstrate the benefits and utility of the novel TMFs and comprehensive reviews comparing them with the more established TMFs such as the BCWl [13]. The CFIR’s emphasis on context informed our consideration of potential contextual variations in DBCI implementation and our suggestion that newly developed TMFs should also consider these contexts, balancing adaptability with specificity to facilitate uptake. Additionally, it would be helpful to have a comprehensive database summarizing the models and their potential use cases.

By critically analyzing potential reasons for the slow uptake of DBCI-specific frameworks, this study has contributed a valuable insight into the existing theory-intervention gap and provided recommendations to bridge this gap. The study thus supports and expands on findings from previous research on the applicability and utility of these TMFs [3, 9]. It is important to note that while our critical analysis of the TMFs and their citations highlights several challenges with the new frameworks, their lack of adoption may be because they are quite recent, and might increase in the coming years. However, the barriers identified in this commentary (limited empirical support, inconsistent reporting, and difficulties in operationalization) may prevent widespread uptake without targeted efforts to mitigate them. The potential of the novel TMFs to address the unique demands specific to digital health settings may therefore remain unrealized without clearer guidance and stronger evidence, in contrast to established models such as the BCW, which may not fully address the challenges of digital contexts but benefit from broad recognition and well-documented application. Limitations common to all commentaries apply, namely that while this perspective is based on the literature, it is not linked to primary research that we have conducted.

This study has highlighted several avenues for future research, including suggestions for TMF developers to enhance the uptake of their own frameworks, such as providing detailed strategies for their use in intervention design, ensuring that the TMFs are adaptable, and generating preliminary evidence of their utility. Our recommendations are consistent with previous qualitative research, which suggested improvements in TMF accessibility and reporting [9]. The novel models we have referenced have contributed great insights and have the potential to improve DBCI design. It is encouraged to acknowledge the evolution of TMFs, particularly while developing digital interventions, for meaningful behavior change in the future.

## Welfare of Animals

This article does not contain any studies with animals performed by any of the authors.
